# Multidrug-Resistant Bacteria and *Enterobacteriaceae* Count in Abattoir Wastes and Its Receiving Waters in Limbe Municipality, Cameroon: Public Health Implications

**DOI:** 10.1155/2022/9977371

**Published:** 2022-03-31

**Authors:** Seraphine Nkie Esemu, Tendongmo Kinsley Aka, Achah Jerome Kfusi, Roland Ndip Ndip, Lucy Mande Ndip

**Affiliations:** ^1^Laboratory for Emerging Infectious Diseases, University of Buea, PO Box 63, Buea, Cameroon; ^2^Department of Microbiology and Parasitology, University of Buea, PO Box 63, Buea, Cameroon

## Abstract

The release of untreated wastes from abattoirs into the environment and nearby water bodies poses a significant threat to public health. Such litters may contain pathogens, including antibiotic-resistant bacteria. This study investigated 80 samples collected from butchering tables, slaughter slabs, meat rinsing points, and abattoir wastes receiving water from two abattoirs (A and B). Total *Enterobacteriaceae* count (TEC) for each sample was determined, and *Escherichia coli* (*E*. *coli*), *Salmonella* spp., *Shigella* spp., and *Staphylococcus aureus* (*S. aureus*) were isolated and identified. Antimicrobial susceptibility testing was done on all bacterial isolates against nine locally used antibiotics. Overall, 118 bacterial isolates, comprising *E. coli* (42.5%), *Salmonella* spp. (27.5%), *Shigella* spp. (37.5%), and *S. aureus* (40.0%), were recovered. Of the 118 bacterial isolates, 104 (88.1%) were multidrug-resistant, including 58 (55.8%) from abattoir A and 46 (44.2%) from abattoir B; however, this difference was not statistically significant (*p* = 0.6837). Of the 32 *S*. *aureus* isolates, 29 (90.6%) were multidrug-resistant. All *S*. *aureus* were 100% sensitive to vancomycin, kanamycin, and amikacin. Similarly, 31 (91.2%) of the 34 *E*. *coli* isolates recovered in this study were multidrug-resistant. *Salmonella* spp. and *Shigella* spp. also showed high levels of multidrug resistance corresponding to 81.8% and 86.7%, respectively. All isolates of *E. coli*, *Salmonella*, and *Shigella* were 100% resistant to ampicillin and 100% sensitive to ciprofloxacin. Minimum and maximum mean values for TEC were 3.62-5.83 log CFU/mL for abattoir A and 4.08–5.56 log CFU/mL for abattoir B. The highest and lowest TEC counts were from slaughter slab and upstream water, respectively, in each abattoir. Our results indicate a predominance of multidrug-resistant bacteria in abattoir wastes and their receiving waters in the study sites. Hence, we recommend the treatment of abattoir wastes before disposal and improved hygiene and sanitation practices to enhance public health.

## 1. Introduction

Functional adequate abattoir wastes management systems and policies are almost inexistent in many African countries. This gap leads to the unhygienic disposal of solid wastes and untreated effluent into the environment [1]. Wastes are either disposed of in open dumps or discharged into nearby streams, constituting an environmental and public health menace [2, 3]. Proper waste management in the abattoir is critical in ensuring public health and environmental safety [4]. The risk of epidemics, water contamination, and pollution are real problems confronting developing countries where abattoir waste management issues are grossly neglected [5, 6]. Untreated wastewater from abattoirs reportedly contains high levels of total coliform bacteria beyond the levels recommended for discharge into water bodies [7]. Pathogenic bacteria such as *E. coli*, *Salmonella*, and *Shigella* spp. have been detected in abattoir settings [6].

Abattoirs have been recognized as a critical link in spreading pathogenic bacteria to the environment [8, 9], including multidrug-resistant pathogenic organisms capable of causing difficult-to-treat infections in humans and animals [10]. Antibiotic-resistant bacteria (ARB) in abattoirs has been the topic of numerous international health and political summits. An abundance of comprehensive reports, guidelines, and recommendations at international and national levels have been published to tackle the threats posed by antibiotic resistance [11]. Several studies have reported the presence of antibiotic-resistant bacteria in abattoir wastes [12–14], and among these bacteria, multidrug-resistant *Salmonella* [13] and enterohemorrhagic *E*. *coli* (EHEC) 0157:H7 [15] were detected. *S. aureus* and methicillin-resistant *S*. *aureus* have also been reported from the abattoir environment [16]).

Antibiotic resistance has recently been referred to as “the silent tsunami facing modern medicine” [17, 18]. Even ARB that are nonpathogenic and part of the normal intestinal flora have been shown to transfer resistance genes to pathogenic bacteria such as *Salmonella* and EHEC O157:H7 [13]. *Salmonella* spp. and *E. coli* are examples of zoonotic bacteria known to cause diseases in humans and could be present in high levels in abattoir wastes [5]. Nwanta et al. [13] examined abattoir wastes for bacteria with potential risk for human health at an abattoir in Nigeria. They identified several bacteria, including *E. coli* O157:H7, *Salmonella* spp. and *Campylobacter* species. Another study reported many pathogenic microorganisms such as *Salmonella*, *E. coli* (including serotype 0157:H7), *Shigella*, parasite eggs, and amoebic cysts [1], which are of public health importance. Another earlier study isolated pathogenic bacteria and fungi species from abattoir wastewater and surface water, including *Staphylococcus*, *E. coli*, *Streptococcus*, *Salmonella*, *Aspergillus*, *Mucor*, *Saccharomyces*, and *Penicillium* species [19].

Evaluating antimicrobial susceptibility profiles of various bacterial pathogens in abattoir waste and their receiving waters is critical to assess the potential risk of disseminating resistant pathogens to the environment and the human population. There is limited information on antimicrobial susceptibility profiles of bacterial pathogens in abattoir wastes and their receiving waters in Limbe municipality and Cameroon in general. This study assessed the total *Enterobacteriaceae* count and antimicrobial susceptibility patterns of common bacterial pathogens isolated from abattoir wastes.

## 2. Materials and Methods

### 2.1. Study Sites

**Figure 1:**
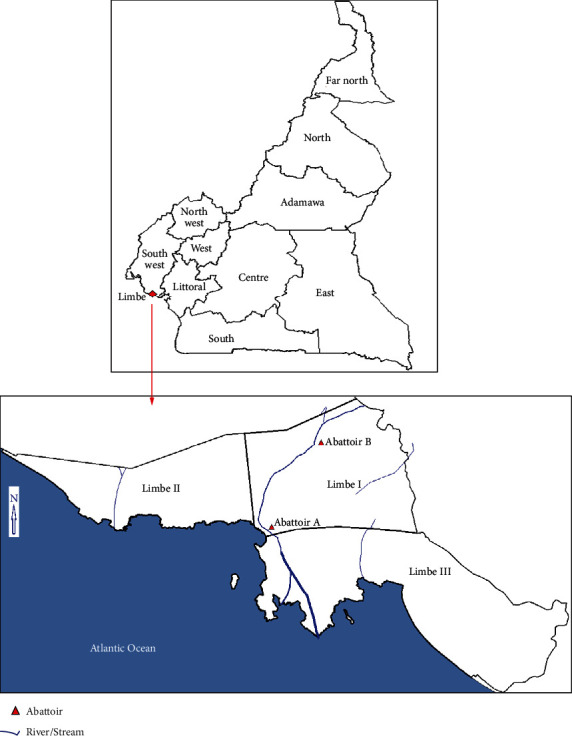
Map of Limbe showing abattoirs A and B.

This study was carried out in two abattoirs (designated A and B) in Limbe (Figure 1), Fako Division, South West Region of Cameroon. Limbe (4.024100, 9.214800) is a coastal town situated at the foot of Mount Cameroon and an international tourist destination. Its main touristic features are beautiful coastal beaches, historical monuments, a botanic garden, and a wildlife center. Limbe had over 120,000 inhabitants in 2012, with an estimated growth rate of 2.9% [20]. It has a surface area of 545 km^2^ and one of the highest population densities in Cameroon, with 220 people per km^2^ [20].

Abattoir A (4.008900, 9.214700) is the central functional abattoir. This abattoir has been in existence for over 70 years, and an average of 85 cattle (range 50 to 120) are slaughtered daily. Abattoir B (4.0602, 9.2447), relatively smaller, with an average slaughtering capacity of 37 cattle per day, is approximately 7 km from abattoir A. This abattoir was constructed in 2014 to reduce overcrowding at the central abattoir.

### 2.2. Sample Collection and Transportation

This study was a laboratory-based investigation, and samples were collected weekly from each abattoir for eight weeks. Swab samples were collected from the slaughter slab and butchering table, while water samples were collected from the meat rinsing point and the abattoir wastes receiving waters. An electrostatic swab cloth was rubbed over different points on the surfaces of varying butchering tables in the swab collection procedure. The fabric was immediately immersed in a sterile sample collection bottle containing 5 mL of sterile phosphate-buffered saline. A sampling of the wastes receiving waters was done upstream and downstream; equidistant (100 m) from the abattoir wastes discharge point into the stream. Overall, 80 samples (40 from each abattoir) were collected. All samples were maintained at a temperature of 4°C in a cool box with ice packs to prevent the multiplication of endogenous microbes. Samples were transported to the Laboratory for Emerging Infectious Diseases, at the University of Buea, within 2 h of collection for analysis. On the first day of sample collection, ancillary data, including the average number of cattle slaughtered per day, age of abattoir, waste disposal methods, presence of water, disinfection of working equipment, and dressing attire, were captured on a simple questionnaire.

### 2.3. Enumeration of *Enterobacteriaceae*

All samples were analyzed for *Enterobacteriaceae* following the British National Standard Method [21]. Aseptically, tenfold serial dilution, up to 10^−7^, was made from 1 mL of each sample and 9 ml of buffered peptone water. From the 10^−5^ to 10^−7^ dilutions, 0.1 mL of the suspension was inoculated by spreading on violet red bile glucose agar in duplicate plates and incubated aerobically at 37°C for 24 h. After incubation, plates containing not more than 150 typical *Enterobacteriaceae* colonies (pink or red, a diameter of 0.5 mm or greater, and with or without precipitation) were counted. For each sample, five of the colonies counted as *Enterobacteriaceae* were selected randomly, subcultured onto nutrient agar (CM131, Oxoid, USA), and incubated overnight at 37°C. Colonies were confirmed as *Enterobacteriaceae* by observation of glucose fermentation and adverse oxidase reaction. Results were obtained as the average count in duplicate plates and expressed as CFU/mL of the sample using the following formula.

(1) $$\begin{array}{c} \text{Count per mL}=\frac{\text{Number of colonies confirmed}}{\text{Number of colonies tested}}\;\times \frac{\text{Number of colonies counted}}{\text{Volume tested}\times \text{Dilution}}. \end{array}$$

The *Enterobacteriaceae* counts were transformed to log_10_ CFU/mL.

### 2.4. Isolation and Identification of Bacterial Isolates

#### 2.4.1. *Escherichia coli*

A 200 *μ*L of each sample from the 10^−2^ dilution was inoculated by spreading on MacConkey's agar (Liofilchem, Italy) for the selective and differential isolation of *E. coli* and plates incubated aerobically at 37°C for 24 h. Lactose-fermenting colonies (pink) were Gram-stained and examined microscopically to determine cell morphology and staining reaction. Downstream tests to identify *E*. *coli* included subculture on eosin-methylene blue agar for production of greenish metallic sheen and the miniaturized biochemical tests using API 20E kit (BioMérieux, UK) following purification of presumptive isolates on nutrient agar.

#### 2.4.2. *Salmonella* and *Shigella* Species

To isolate *Salmonella* and *Shigella* species, 200 *μ*L of each undiluted enriched sample were inoculated onto *Salmonella-Shigella* agar (Liofilchem, Italy). Before the inoculation of the SS agar, an aliquot (1 mL) of each sample was enriched in selenite cystine broth (Liofilchem, Italy) in overnight incubation at 37°C to enhance the recovery of *Salmonella* and *Shigella* species. Presumptive isolates were purified on nutrient agar and screened by Gram-staining, motility testing, and the API 20E identification system.

#### 2.4.3. *Staphylococcus aureus*

Similarly, *S. aureus* was isolated on mannitol salt agar (Liofilchem, Italy), a selective and differential culture medium, by inoculation of 200 *μ*L of each undiluted sample. Presumptive *S*. *aureus* isolates were purified on nutrient agar, Gram-stained, and tested for catalase and coagulase production. Confirmation of *S*. *aureus* identity was done using molecular methods. The genomic DNA of each presumptive *S*. *aureus* isolate was extracted using the simple boiling method. Pure colonies of *S*. *aureus* isolates were inoculated into 200 *μ*L of tryptone soy broth (Merck, Darmstadt, Germany) and cultivated overnight at 37°C. The cells were harvested by centrifugation and suspended in 200 *μ*L sterile physiological buffered saline. The suspension was heated in a water bath (Yamato Scientific, USA) at 100°C for 15 min and immediately chilled on ice. The boiled bacterial cells were centrifuged at full speed for 10 min in a microcentrifuge to separate the cell debris from the supernatant. The supernatant was stored at –20°C until used as DNA template.

### 2.5. Polymerase Chain Reaction Identification Using *S. aureus* Species-Specific Primers

All polymerase chain reaction (PCR) amplification reactions were carried out in a total volume of 25 *μ*L comprising 12.5 *μ*L of 2× BioMix master mix (Bioline, USA), forward and reverse primers (0.50 *μ*L each to give a final concentration of 0.4 M), nuclease-free water (6.5 *μ*L), and DNA template (5.0 *μ*L). A negative control was included in each PCR run, in which the DNA template was replaced with nuclease-free water. All PCR runs were carried out using a MyCycler Thermal Cycler (Applied Biosystems, USA). The PCR primer pair F : 5′ − GCGATTGATGGTGATACGGTT − 3′ and R : 5′ − AGCCAAGCCTTGACGAACTAAAGC − 3′ was used to amplify a 280 bp fragment of the thermonuclease (*nuc*) gene of *S*. *aureus* [22]. The PCR cycling conditions were optimized at 94°C for 5 min for one cycle of initial denaturation. This was followed by 35 cycles of denaturation at 94°C for 1 min, annealing at 49°C for 1 min, and extension at 72°C for 1 min. The final extension was set at 72°C for 5 min and cooled to 4°C until tubes were removed from the PCR machine. The PCR products were electrophoresed using 1.5% agarose gel (BioShop, Canada) stained with 0.5 mg/L ethidium bromide (Merck, Modderfontein, South Africa) at 100 V for 1 h, in 1 × TBE buffer and viewed under a UV transilluminator (EBOX VX5, Vilber Lourmat, France).

### 2.6. Antibiotic Susceptibility Testing

All confirmed isolates of *E*. *coli*, *Salmonella* spp., *Shigella* spp., and *S*. *aureus* were subjected to *in vitro* susceptibility testing against commonly used antimicrobial agents using the Kirby-Bauer disc diffusion method and following guidelines established by the Clinical and Laboratory Standards Institute [23]. The antibiotics used, their potency and abbreviations, were as follows: ampicillin (10 *μ*g, AM), amoxicillin-clavulanate (10 *μ*g, AMC), penicillin (10 *μ*g, P), nalidixic acid (30 *μ*g, NA), tetracycline (30 *μ*g, TE), streptomycin (30 *μ*g, S), gentamicin (10 *μ*g, GM), amikacin (30 *μ*g, AK), kanamycin (30 *μ*g, K), chloramphenicol (10 *μ*g, C), vancomycin (30 *μ*g, VA), ceftriaxone (30 *μ*g, CRO), clindamycin (2 *μ*g, DA), and ciprofloxacin (5 *μ*g, CIP). These antibiotics were chosen because they are used in human medicine and/or animal veterinary practice in the study area. After incubation, the diameter of the zones of inhibition around each disc was measured. These diameters were interpreted as resistant, intermediate, or sensitive following the Clinical Laboratory Standards Institute [23].

### 2.7. Statistical Analysis

*Enterobacteriaceae* counts were transformed to log10 CFU/mL before statistical analysis. Data generated on the prevalence of bacterial isolates and multidrug-resistant bacterial isolates were analyzed using a chi-squared test to determine whether there were significant differences in the prevalence of these isolates and *Enterobacteriaceae* counts between abattoirs and between sample collection points. Statistical significance was set at a *p* value of <0.05. Charts were plotted using Microsoft Excel 2010.

## 3. Results

### 3.1. Characteristics of the Abattoirs

**Figure 2:**
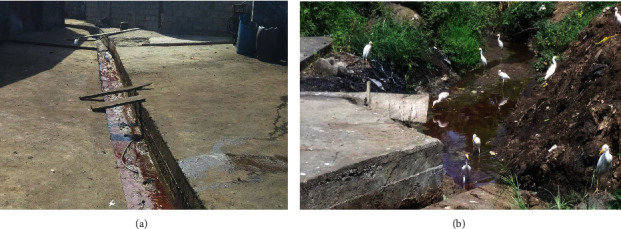
Abattoir A environment: (a) drainage designed to channel liquid wastes to the environment and nearby stream; b) heap of cattle dung frequented by cattle egrets.

From information captured in the questionnaire, the two abattoirs differed only at the level of the number of cattle slaughtered per day and their longevity (Table 1). Both abattoirs undertook similar wastes disposal methods characterized by lack of waste treatment and no disinfection of slabs, tables, and equipment (Table 1). Information collected from direct observation of the abattoir environment confirmed that the hygiene status of both abattoirs was poor. Slow-moving abattoir effluents that trickled through purposely designed drainages to the abattoir surroundings and subsequently to the receiving water were observed (Figure 2(a)). On each sample collection day, flies, rodents, other potential disease-carrying vectors, and cattle egrets were seen on heaps of wastes (mainly cattle dung and unused parts) in the vicinity of the abattoirs (Figure 2(b)).

The abattoirs had no toilet facilities, and unlike abattoir B, residential buildings were very near abattoir A. It was also observed that proper regular cleaning of the floor with disinfectants was not done. Still, more animals were brought and slaughtered in the exact location containing large quantities of blood and animal waste.

The slaughter slabs in both abattoirs were tiled, and the tiles had several coats of dirt, suggesting they were not washed regularly. All animals were slaughtered on the same slab. The butchering block (referred to as the butchering table in this study) in abattoir A was not smooth because several tiles had fallen off. Although tap water was present in both abattoirs, the water used at the meat rinsing points was held in drums and was rarely changed.

### 3.2. Total *Enterobacteriaceae* Count

**Figure 3:**
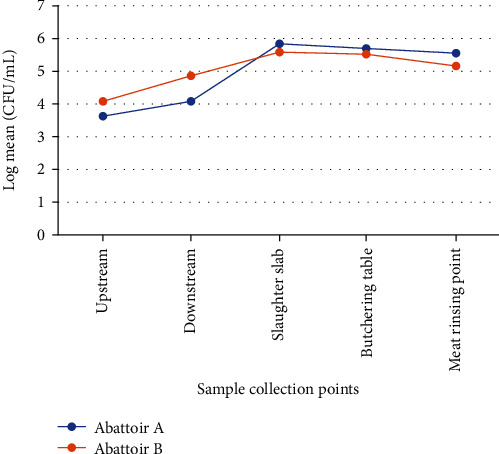
Total *Enterobacteriaceae* counts at each sample collection point.

The mean values of the *Enterobacteriaceae* count for each abattoir were computed and are presented in Table 2. The typical contamination sites in both abattoirs were the slaughter slabs, the butchering tables, and the meat rinsing points. The counts were lowest for upstream samples (abattoir A, 2.0 × 10^3^ − 1.0 × 10^4^ CFU/mL; abattoir B, 4.2 × 10^3^ − 2.0 × 10^4^ CFU/mL) and highest for samples from the slaughter slab (abattoir A, 5.8 × 10^5^ − 8.0 × 10^5^ CFU/mL; abattoir B, 1.8 × 10^5^ − 5.5 × 10^5^ CFU/mL) (Table 2 and Figure 3). There was no significant difference in mean *Enterobacteriaceae* numbers from the different sampling points between abattoirs A and B (*p* > 0.05) except for that between upstream and downstream for abattoir A (*p* = 0.02) and abattoir B (*p* = 0.03).

### 3.3. Prevalence of Bacterial Isolates

A total of 118 bacterial isolates were identified as *E*. *coli* (*n* = 34; 28.8%), *Salmonella* spp. (*n* = 22; 18.6%), *Shigella* spp. (*n* = 30; 37.5%), and *S*. *aureus* (*n* = 32; 40.0%). Of the 118 bacterial isolates, 55.1% (65/118) were from abattoir A and 45.0% (53/118) from abattoir B (Table 3).

### 3.4. Antibiotic Susceptibility Pattern of Bacterial Isolates

Each bacterial isolate was challenged with a panel of nine antibiotics. The Gram-negative rods (*E. coli*, *Salmonella* spp., and *Shigella* spp.) were challenged with the same antibiotics. In contrast, *S*. *aureus* (Gram-positive cocci) was challenged with antibiotics which included penicillin, amikacin, kanamycin, clindamycin, and vancomycin (Table 4). The antibiotic resistance pattern showed that all isolates of *E. coli*, *Salmonella* spp., and *Shigella* spp. were resistant to ampicillin and also showed high resistance to ceftriaxone. All the isolates of *E. coli*, *Salmonella* spp., and *Shigella* spp. were sensitive to ciprofloxacin. The *E*. *coli* isolates had the least resistance to nalidixic acid (8.8%, 3/34) followed by *Salmonella* spp. 59.1% (13/22) and *Shigella* spp. with zero resistance (Table 4). All *S*. *aureus* isolates were sensitive to amikacin, kanamycin, and vancomycin. On the contrary, *S*. *aureus* isolates were 100% resistant to penicillin, followed by ceftriaxone (84.4%, 27/32) and tetracycline (65.6%, 21/32) (Table 4).

### 3.5. Multidrug Resistance Patterns of Bacterial Isolates

**Figure 4:**
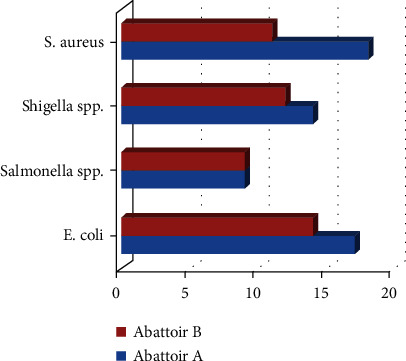
The proportion of multidrug-resistant isolates in abattoirs A and B.

Unfortunately, of the 118 bacterial isolates challenged with antibiotics, 88.1% (104/118) elaborated multidrug resistance (resistance to at least one antibiotic in three or more classes of antibiotics). The 104 multidrug-resistant isolates comprised 58 (55.8%) from abattoir A and 46 (44.2%) from abattoir B; however, this difference was not statistically significant (Table 5 and Figure 4). Except for the prevalence of *Salmonella* spp. that was the same in both abattoirs, the prevalence of other multidrug-resistant bacterial isolates was higher in abattoir A than abattoir B. However, the difference was not statistically significant.

There was considerable variation in the prevalence of multidrug-resistant isolates from different sample collection points. The highest prevalence of multidrug-resistant bacteria was downstream from the point of discharge of the effluents in abattoir A, followed by the slaughter slab in abattoir A, meat rinsing point in abattoir A, and butchering table in abattoir A. No multidrug-resistant *Shigella* spp. and *S. aureus* were recovered upstream of abattoir B (Table 6).

### 3.6. Antibiotypes of Bacterial Isolates Circulating in the Study Sites

A total of 50 antibiotypes (designated L1–L44) were identified. The 50 antibiotypes comprised 14 from the 34 *E*. *coli* isolates, 12 from the 22 *Salmonella* spp., 11 from the 30 *Shigella* spp. and 13 from the 32 S. *aureus* isolates (Table 7). The most prevalent antibiotype pattern was L32, and it was typical in six *Shigella* isolates, while L11 and L35 had five *Shigella* and *S*. *aureus* isolates, respectively.

## 4. Discussion

Antibiotic resistance has recently been referred to as “the silent tsunami facing modern medicine” [17, 18] and also a “One Health” challenge due to the rapid emergence and spread of resistant bacteria among humans, animals, and the environment [24, 25]. Abattoir wastes and effluents are considered a hotspot for antibiotic-resistant bacteria and are thought to play an essential role in disseminating the antibiotic-resistant bacteria into the environment as well as the human and animal populations [26]. Therefore, constant monitoring and intermittent microbial analysis of the abattoir environment are necessary to maintain hygienic conditions and curb the spread of pathogens, including antibiotic-resistant bacteria [7]. Tracking antibiotic resistance is vital in identifying high-risk environments, especially in developing countries like Cameroon, where available data on antibiotic resistance is minimal. Ecological niches, such as abattoir wastes and their receiving waters that are nutrient-rich and characterized by high bacterial concentrations, are ideal environments for developing and disseminating antibiotic-resistant bacteria and require constant monitoring. Therefore, this study has colossal importance as it extends our knowledge regarding the extent of the antimicrobial resistance menace. Additionally, this type of study that reveals the extent of the antibiotic resistance issue could influence the think tanks to peruse the matter urgently [27].

An abattoir (also called a slaughterhouse) is a premise approved and registered by the controlling authority for hygienic slaughtering and inspection of animals, processing and practical preservation, and storing meat products for human consumption [28]. Therefore, the application of good hygiene practices at abattoirs is essential for improving both meat quality and public health [29]. Abattoirs produce enormous amounts of wastes, and in most developing countries, the improper disposal of the trash has made these abattoirs a source of embarrassment and an ecological calamity [27]. Abattoir wastes have been reported to contain several pathogens, including antibiotic-resistant bacteria. Hence, unhygienic practices and poor sanitation at abattoirs constitute a significant driver of antibiotic resistance [26, 27]. Improved awareness and understanding of antimicrobial resistance problems through effective communication and educational programs on hygiene and health are necessary to fight antibiotic resistance [26, 27, 30]. Good sanitation and hygiene can slow the spread of antibiotic-resistant bacteria from abattoirs, thereby preventing the emergence of difficult-to-treat antibiotic-resistant infections [30].

In this study, the hygiene of the abattoir environment was assessed by a microbiological criterion involving *Enterobacteriaceae* count. Our results showed high levels of *Enterobacteriaceae* at all sample collection points and for each sampling day ranging from 3.62 Log CFU/mL (upstream) to 5.83 Log CFU/mL (slaughter slab) for abattoir A and from 4.08 Log CFU/mL (upstream) to 5.56 Log CFU/mL (slaughter slab) for abattoir B. The higher EC recorded from the slaughter slab in abattoir A could be due to overcrowdedness, increased abattoir activities, and the generation of more wastes and effluents. However, this difference was not statistically significant (*p* > 0.05). These results suggest that the management of the abattoirs does not implement good hygiene and sanitation practices, which compromises the quality of animal carcasses and public health [29]. Although strict hygiene rules are recommended for abattoir activities, no legal limits or reduction levels have been fixed for microbiological contamination of wastewater in Cameroon. Since regular cleaning and disinfection of surfaces was not done in either abattoir, there was probably a build-up of contamination on surfaces and equipment in the slaughter slab, butchering table, and meat rinsing points. So far, EC is very relevant for the proper identification and evaluation of abattoir hygiene [31] and evaluation of preslaughter environments [32, 33].

The high EC mirrored the presence of bacterial pathogens in the abattoir wastes and their receiving waters. The 118 confirmed bacterial isolates comprised *E. coli* (34, 42.5%), *Salmonella* spp. (22, 27.5%), *Shigella* spp. (30, 37.5%), and *S. aureus* (32, 40.0%). These microorganisms are problematic mainly because they are human pathogens and are also incriminated in foodborne diseases. These pathogens have been identified in the abattoir environment elsewhere [7, 34–36]. Based on the number of samples analyzed, the prevalence of *E. coli* observed in this study in both abattoirs (abattoir A: 45%, 18/40; abattoir B: 40%, 16/40) was higher than reported from Mojo, Ethiopia (23%) [37], and slightly lower than that from an abattoir in Botswana (62.3%) [38]. A lower prevalence of *Salmonella* spp. was reported in this study (abattoir A: 30%, 12/40; abattoir B: 25%, 10/40) than reported in Modjo abattoir in Ethiopia (89%) [37], 33.3% in Ogbete abattoir in Enugu State, Nigeria [39], and 19.5% reported in Sokoto abattoir in Nigeria [40].

All isolates of *E. coli*, *Salmonella*, and *Shigella* spp. were 100% resistant to ampicillin, followed by ceftriaxone which had 88.2%, 81.8%, and 60.0% for *E*. *coli*, *Salmonella*, and *Shigella* isolates, respectively (Table 4). These isolates showed 100% sensitivity to ciprofloxacin, followed by nalidixic acid with 100% (*Shigella* spp.) and 91.2% for *E*. *coli*. Amoxicillin-clavulanate effectively killed the bacterial isolates with 93.3%, 88.2, and 77.3% for *Shigella*, *E*. *coli*, and *Salmonella* isolates, respectively. All *S*. *aureus* were 100% sensitive to vancomycin, kanamycin, and amikacin. High susceptibility to ciprofloxacin, amoxicillin-clavulanate, and nalidixic acid has been recorded from previous studies conducted in Ethiopia and Nigeria [36].

Antimicrobial resistance in these bacterial agents is increasing worldwide, and its susceptibility patterns show substantial geographic variation and differences in population and environment [41]. Surprisingly, of the 118 bacterial isolates, 104 (88.1%) were multidrug-resistant, including 58 (55.8%) from abattoir A and 46 (44.2%) from abattoir B; however, this difference was not statistically significant (*p* = 0.6837). Multidrug-resistant bacteria have an enhanced capacity for surviving and thriving in their host and surrounding environment in the presence of several antimicrobial agents [42]. Persons infected with multidrug-resistant isolates have much higher death rates and increased complications and suffering [43]. Of the 32 *S. aureus* isolates, 29 (90.6%) were multidrug-resistant. Similarly, 31 (91.2%) of the 34 *E*. *coli* isolates recovered in this study were multidrug-resistant. *Salmonella* spp. and *Shigella* spp. also showed high levels of multidrug resistance corresponding to 81.8% (18/22) and 86.7% (26/30), respectively. The high rate of multidrug resistance recorded in this study is consistent with the results of Ventola [44].

## 5. Conclusions

Our study revealed several bacterial pathogens in abattoir waste and its receiving waters, most of which were resistant to commonly used antimicrobials. The majority of these pathogens were multidrug-resistant. These pathogens were released to the environment and nearby water bodies through poor hygiene and sanitation practices, posing significant public health threats. The results of this study add to the mounting evidence that abattoirs constitute a substantial link in the spread of antibiotic resistance.

## Figures and Tables

**Table 1:** Ancillary data on the primary activities at the two abattoirs.

Characteristic	Abattoir A	Abattoir B
The average number of cattle slaughtered per day	85	37
Age of abattoir (years)	80	8
Method of solid wastes disposal	Burning/dumping	Burning/dumping
Method of liquid wastes disposal	Nearby stream	Nearby stream
Treatment of waste before disposal	No	No
Disinfection of slabs and tables	No	No
Disinfection of equipment	No	No
Presence of tap water	Yes	Yes
Wearing protective equipment (coats, gloves, boats)	Occasionally	Occasionally

**Table 2:** Total *Enterobacteriaceae* counts from each abattoir and sample collection point.

Abattoir	Sample collection point	Number of samples analyzed	Counts (CFU/mL)			
			Minimum	Maximum	Mean	Log mean
A	Upstream	8	2.0 × 10^3^	1.0 × 10^4^	4.2 × 10^3^	3.62
	Downstream	8	6.6 × 10^3^	8.8 × 10^4^	1.2 × 10^4^	4.08
	Slaughter slab	8	5.8 × 10^5^	8.0 × 10^5^	6.8 × 10^5^	5.83
	Butchering table	8	2.5 × 10^5^	6.7 × 10^5^	4.8 × 10^5^	5.68
	Meat rinsing point	8	1.6 × 10^5^	4.1 × 10^5^	3.5 × 10^5^	5.54
B	Upstream	8	4.2 × 10^3^	2.0 × 10^4^	1.2 × 10^4^	4.08
	Downstream	8	6.1 × 10^4^	1.5 × 10^5^	7.2 × 10^4^	4.86
	Slaughter slab	8	1.8 × 10^5^	5.5 × 10^5^	3.6 × 10^5^	5.56
	Butchering table	8	1.5 × 10^5^	6.1 × 10^5^	3.2 × 10^5^	5.51
	Meat rinsing point	8	1.1 × 10^5^	2.5 × 10^5^	1.5 × 10^5^	5.17

**Table 3:** Bacterial isolates identified in the study sites.

Bacterial species	Total isolated (%)	Abattoir (*n* = 40 each)	
		A (%)	B (%)
*E*. *coli*	34 (28.8)	18 (15.3)	16 (13.6)
*Salmonella* spp.	22 (18.6)	12 (10.2)	10 (8.5)
*Shigella* spp.	30 (25.4)	15 (12.7)	15 (12.7)
*S*. *aureus*	32 (27.1)	20 (16.9)	12 (10.2)
Total	*118 (99.9)*	*65 (55.1)*	*53 (45.0)*

**Table 4:** Bacterial isolates resistant to each antibiotic tested.

Class of antibiotic	Antibiotic	Number (%) of resistant bacterial isolates			
		*E. coli* (*N* = 34)	*Salmonella* spp. (*N* = 22)	*Shigella* spp. (*N* = 30)	*S. aureus* (*N* = 32)
Penicillins	Ampicillin (AM)	34 (100)	22 (100)	30 (100)	—
	Amoxicillin-clavulanate (AMC)	4 (11.8)	5 (22.7)	2 (6.7)	—
	Penicillin (P)	—	—	—	32 (100)
Quinolone	Nalidixic acid (NA)	3 (8.8)	13 (59.1)	0	—
Tetracyclines	Tetracyline (TE)	8 (23.5)	17 (77.3)	8 (26.7)	21 (65.6)
Aminoglycoside	Streptomycin (S)	20 (58.8)	15 (68.2)	15 (50.0)	—
	Gentamicin (GM)	15 (44.1)	11 (50.0)	10 (33.3)	11 (34.4)
	Amikacin (AK)	—	—	—	0
	Kanamycin (K)	—	—	—	0
Phenicols	Chloramphenicol (C)	14 (41.2)	9 (40.9)	17 (56.7)	—
Glycopeptide	Vancomycin (VA)	—	—	—	0
Cephalosporins	Ceftriaxone (CRO)	30 (88.2)	18 (81.8)	18 (60.0)	27 (84.4)
Lincosamides	Clindamycin (DA)	—	—	—	9 (28.1)
Fluoroquinolones	Ciprofloxacin (CIP)	0	0	0	5 (15.6)

—, Not done.

**Table 5:** Distribution of the multidrug-resistant isolates in abattoirs A and B.

Bacterial species	Multidrug-resistant isolates (%)			*p*-value
	Total (%)	Abattoir A (%)	Abattoir B (%)	
*E*. *coli*	31 (29.8)	17 (54.8)	14 (45.2)	0.4761
*Salmonella* spp.	18 (17.3)	9 (50.0)	9 (50.0)	
*Shigella* spp.	26 (25.0)	14 (53.8)	12 (46.2)	0.2827
*S*. *aureus*	29 (27.9)	18 (62.1)	11 (37.9)	0.8756
Total	*104 (100)*	*58 (55.8)*	*46 (44.2)*	0.6837

**Table 6:** Distribution and prevalence of multidrug-resistant bacterial isolates.

Multidrug-resistant bacterial species	Prevalence of multidrug-resistant bacterial isolates (%)										Total
	Upstream		Downstream		Slaughter slab		Butchering table		Meat rinsing point		
	Abattoir A	Abattoir B	Abattoir A	Abattoir B	Abattoir A	Abattoir B	Abattoir A	Abattoir B	Abattoir A	Abattoir B	
*E*. *coli*	2 (1.9)	3 (2.9)	4 (3.8)	4 (3.8)	4 (3.8)	2 (1.9)	3 (2.9)	3 (2.9)	3 (2.9)	3 (2.9)	*31 (29.8)*
*Salmonella* spp.	2 (1.9)	1 (1.0)	3 (2.9)	2 (1.9)	2 (1.9)	2 (1.9)	1 (1.0)	2 (1.9)	1 (1.0)	2 (1.9)	*18 (17.3)*
*Shigella* spp.	2 (1.9)	0 (0.0)	3 (2.9)	3 (2.9)	4 (3.8)	3 (2.9)	3 (2.9)	2 (1.9)	4 (3.8)	2 (1.9)	*26 (25.0)*
*S*. *aureus*	1 (1.0)	0 (0.0)	7 (6.7)	5 (4.8)	5 (4.8)	2 (1.9)	3 (2.9)	2 (1.9)	3 (2.9)	1 (1.0)	*29 (27.9)*
*Total*	*7 (6.7)*	*4 (3.8)*	*17 (16.3)*	*14 (13.5)*	*15 (14.4)*	*9 (8.7)*	*10 (9.6)*	*9 (8.7)*	*11 (10.6)*	*8 (7.7)*	*104 (100)*

**Table 7:** Antibiotypes of bacterial isolates identified in this study.

Pattern	Antibiotype	Multidrug-resistant	*E. coli*	*Salmonella* spp.	*Shigella* spp.	*S. aureus*
L1	AM_CRO_NA_TE_GM_C_AMC_S	+		2		
L2	AM_CRO_NA_TE_GM_AMC	+		1		
L3	AM_CRO_NA_GM_AMC_S	+		1		
L4	AM_CRO_GM_C_AMC_S	+	3	1		
L5	P_TE_CRO_CIP_DA_GM	+				4
L6	AM_CRO_NA_GM_C_S	+	2	3		
L7	AM_CRO_TE_GM_C_S	+		2		
L8	AM_CRO_GEN_NA_S	+	2			
L9	AM_CRO_NA_TE_GM	+		1		
L10	P_TE_CRO_CIP_GM	+				2
L11	AM_CRO_C_AMC_S	+			5	
L12	P_TE_CRO_DA_GM	+				4
L13	AM_CRO_TE_C_S	+	4			
L14	AM_TE_GM_C_S	+		2		
L15	AM_NA_GM_C_S	+			3	
L16	AM_CRO_GEN_C	+	3			
L17	AM_CRO_TE_GM	+			1	
L18	AM_CRO_C_GM	+			2	
L19	AM_CRO_NA_TE	+		3		
L20	AM_CRO_GM_S	+	3			
L21	P_TE_CRO_DA	+				1
L22	P_TE_CRO_GM	+				3
L23	P_TE_CIP_GM	+				3
L24	P_TE_CIP_DA	+				2
L25	AM_C_AMC_S	+			3	
L26	AM_TE_GM_S	+			4	
L27	AM_CRO_GM	+	4			
L28	AM_CRO_NA	+		2	1	
L29	AM_CRO_TE	+	3			
L30	AM_TE_C_S	+			1	
L31	AM_CRO_C	+	2			
L32	AM_CRO_S	+			6	
L33	AM_GM_C	+	2			
L34	AM_TE_S	+	3			
L35	P_TE_CIP	+				5
L36	P_CIP_DA	+				3
L37	P_TE_DA	+				2
L38	AM_CRO	—	1	2		
L39	AM_GM	—	1			
L40	P_CRO	—				1
L41	AM_S	—			1	
L42	P_GM	—				1
L43	P_TE	—				1
L44	AM	—	1	2	3	
Total isolates			*34*	*22*	*30*	*32*

Key: AM: ampicillin; AMC: amoxicillin-clavulanate; P: penicillin; NA: nalidixic acid; TE: tetracycline; S: streptomycin; GM: gentamycin; C: chloramphenicol; CRO: ceftriaxone; DA: clindamycin.

## Data Availability

Relevant data that support the findings of this study have been included in this manuscript.

## Abbreviations

AK: Amikacin
AM: Ampicillin
ARB: Antibiotic-resistant bacteria
AMC: Amoxicillin-clavulanate
C: Chloramphenicol
CFU: Colony forming unit
CIP: Ciprofloxacin
CRO: Ceftriaxone
DA: Clindamycin
EHEC: Enterohemorrhagic *E. coli*
GM: Gentamicin
K: Kanamycin
NA: Nalidixic acid
S: Streptomycin
spp.: Species
TE: Tetracycline
UV: Ultraviolet
VA: Vancomycin
TEC: Total *Enterobacteriaceae* count
P: Penicillin
PCR: Polymerase chain reaction.
